# Procedures for determining the antimicrobial activity of maggot excretions and secretions of the green bottle fly: a narrative literature review

**DOI:** 10.1590/1414-431X2025e14851

**Published:** 2025-09-12

**Authors:** D.L. Dallavecchia, A.A. de Lima e Silva, A.C.M. Debelian, V.M. Aguiar, R.G. Silva

**Affiliations:** 1 Laboratório de Estudos de Dípteros, Departamento de Microbiologia e Parasitologia, Instituto Biomédico, Universidade Federal do Estado do Rio de Janeiro, Rio de Janeiro, RJ, Brasil; 2 Laboratório de Microbiologia Aplicada, Departamento de Biologia e CESAM, Universidade de Aveiro, Aveiro, Portugal; 3 Laboratório de Biologia e Fisiologia de Microrganismos, Departamento de Microbiologia e Parasitologia, Instituto Biomédico, Universidade Federal do Estado do Rio de Janeiro, Rio de Janeiro, RJ, Brasil

**Keywords:** Native excretions and secretions (NES), Maggot therapy, Calliphoridae, Antimicrobial activity

## Abstract

The therapeutic benefits obtained from the presence of larvae of certain flies in infected wounds have been mentioned since ancient times. Currently, the so-called maggot therapy or biosurgery is considered a simple, safe, relatively low-cost, and highly effective alternative for treating a wide variety of infected, necrotic, and difficult-to-heal wounds, including those caused by multidrug-resistant bacteria. In addition to the debridement of necrotic wound tissue promoted by larvae, especially from the green bottle fly (*Lucilia sericata*; Diptera: Calliphoridae), there is much evidence that their native excretions and secretions (NES) contain components with varied antimicrobial activity against Gram-positive and Gram-negative bacteria and activity against fungi. Furthermore, studies have shown the antibiofilm effect of NES. Biofilms represent an additional problem for wound healing because they prevent the action of antibiotics on the pathogens infecting the wound. The antimicrobial effects of crude NES or its molecular components described in studies sometimes present contrasting results when compared. This is probably due to the laboratory methodological aspects used, which range from the preparation of larvae and extraction of NES to the tests used to evaluate their antimicrobial activity. This review aimed to bring together a diversity of laboratory procedures and results that have been described for the antimicrobial potency of NES. Moreover, it aimed to contribute to a greater standardization of the methodologies adopted in new studies to generate more consensual and comparable results.

## Introduction

The use of larvae of certain flies to heal infected wounds dates back to ancient cultures (1). However, the first formal medical record of this potentially beneficial effect dates back to 1500 by the French surgeon Ambroise Paré (1,2). Later, several reports involving soldiers on battlefields provided more detailed data on this larval activity (2-4), like those of the surgeon Dominique Jean Larrey in 1829, who accompanied Napoleon Bonaparte on several expeditions and campaigns. While treating wounded soldiers in Syria, he reported that blue fly larvae were not harmful to wounds, but rather promoted their healing. The larvae accelerate the natural process by digesting the cellular eschars (3). In the American Civil War, confederate army surgeon F. Zacharias reported that he applied maggots to remove decayed tissue in hospital gangrene. He concluded that in a single day, they would clean a wound far better than any agents available at the time. Later, Zacharias used this procedure in several places and claimed that it saved many lives (4).

During World War I in 1917, American surgeon William Baer treated two soldiers who had been left on the battlefield for a week after the fight. After cleaning and removing the larvae, Baer (4) observed no pus in the lesions, and the internal structure of the injured bone and the adjacent parts were entirely covered by pink granulation tissue covering the wound. These patients continued to heal, and ten years later, still impressed by the characteristics of these wounds, Baer (then Professor of orthopedic surgery at Johns Hopkins University) decided to test the observations made on the battlefield. In September 1928, he applied unsterilized blowfly larvae to the wounds of four children whose primary treatment for osteomyelitis had failed. After six weeks, the lesions had completely healed, not only the deeper structures, but also the skin. After an interruption of the experiments due to the unavailability of larvae, in April and May 1929, new cases were subjected to this treatment method with the same results. However, cases of secondary infection by *Clostridium tetani* and *C. perfringens* were observed in some patients, and all experiments were interrupted until this difficulty was overcome. Then, Baer concluded that it would be necessary to have sterile larvae. Furthermore, a larvae cultivation system would also be necessary for constant production during winter and summer. Requirements to achieve this included full knowledge of the blowfly life cycle, conditions for its procreation and egg laying, type of feeding of the fly and larvae, and essential factors for its growth, such as sunlight, humidity, and temperature (4).

The use of maggots spread rapidly during the 1930s, particularly in the United States, and William Baer is now recognized as the founder of modern maggot therapy (5). However, in the mid-1940s, this type of therapy quickly declined. In addition to the already widespread use of sulfonamides, other factors contributed to this scenario. These factors included the industrial production of penicillin from 1944 onwards, followed by the discovery of other antimicrobials, with a consequent reduction in the incidence of bone and soft tissue infections, improvement in wound treatment and aseptic techniques, improvement in surgical techniques, the high cost of obtaining medicinal larvae, the discomfort for patients, and the unacceptability of dressings with live larvae compared to more recent alternatives (1,5).

**Figure 1:**
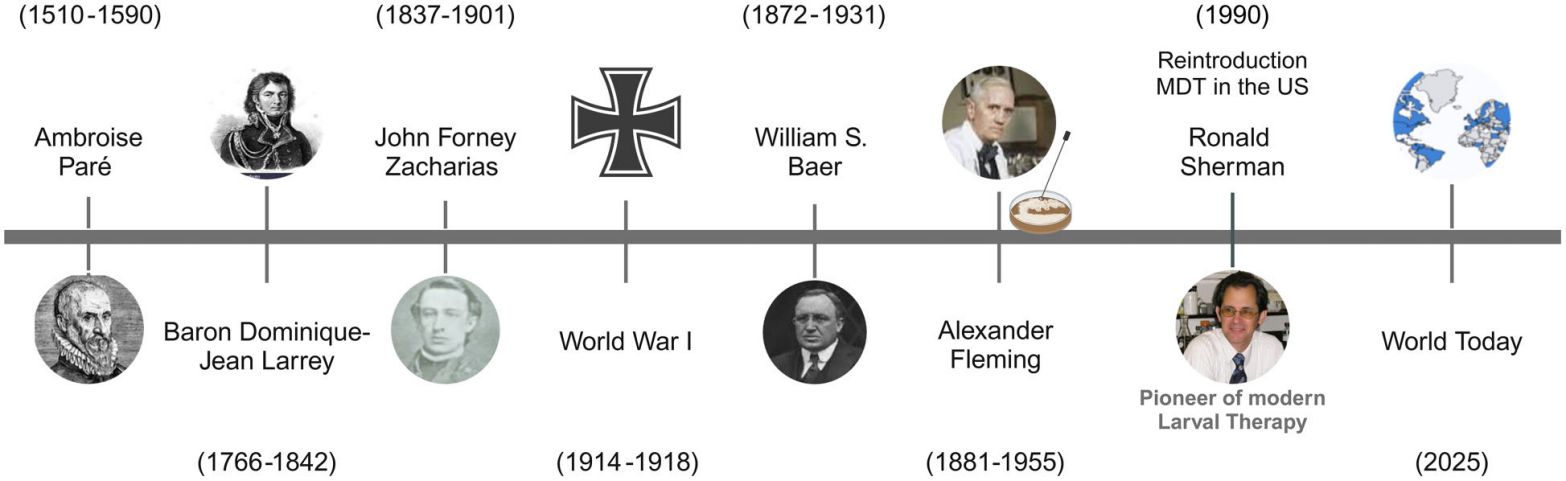
Timeline of maggot debridement therapy (MDT). This figure was kindly provided by Dr. Ronald Sherman.

The 1990s represented a renaissance in maggot therapy, also called biosurgery (6) or larval debridement therapy (7). The renewed interest in this therapeutic practice resulted from factors such as the emergence of multi-drug resistant pathogens, which had a strong impact on chronic wound infections (8), and evidence of the advantages of maggot therapy (Figure 1).

Advantages include high efficacy in debridement of necrotic tissue, safety, and simplicity (5). This renaissance culminated, for example, in the founding of the International Society of Biotherapy in 1996, with the aim of investigating and developing the use of living organisms, or their products, in tissue repair (5). In 2004, the Food and Drug Administration (9) granted authorization for using medicinal maggots as a medical device. Since the larvae's activity is critical to debridement, they have been classified as a medical device rather than a drug (10).

As pointed out by Sherman et al. (5), maggot therapy is essentially a carefully controlled and artificially induced form of myiasis to balance the beneficial effects of larval activity on necrotic tissues (“benign myiasis”) against its potentially negative effects on healthy tissues (“malignant myiasis”). Negative effects may arise, for example, due to the inappropriate use of a species of fly that preferentially feeds on living tissue or the introduction of an excess of larvae into the wound, which can lead to rapid digestion of necrotic tissues, followed by the risk of digesting healthy tissue before the larvae are removed from the wound.

Overall, larval debridement therapy involves placing larvae (mainly of the *Lucilia sericata* (Meigen, 1826) species, one day old and previously sterilized) onto the wound bed of many etiologies. Then, they begin to mechanically debride the necrotic tissue through their oral apparatus while releasing excretions and secretions with substances that can liquefy this tissue, which is easily sucked up by the larvae. This activity helps clean, disinfect, and heal the wound (11,12). This method has been identified as an efficient alternative for dealing with infections by resistant bacteria in difficult-to-heal wounds (10,13-15).

With the deepening of studies on maggot therapy, several pharmacological properties of substances present in the native excretions and secretions (NES) of larvae have been described. These include antibacterial (16), antifungal (17), antiviral (18), anti-tumor (19), anti-biofilm (20), synergistic effects with antibiotics (21), anti-inflammatory activity (22), fibroblast migration, extracellular matrix remodeling (23), and angiogenesis (24). The NES from fly larvae have been widely investigated for their potential action on several bacterial pathogens, including methicillin-resistant *Staphylococcus aureus*, fungi, and even viruses (17,18,25).

Considering the data reported in the literature, maggot therapy is undoubtedly a feasible and advantageous alternative when treating chronic wounds. However, discrepancies and contradictory results have been found regarding the *in vitro* activity of NES, probably due to factors such as differences in experimental methods and conditions under which larvae were prepared. Therefore, this review aimed to systematize and discuss the procedures used to obtain NES and describe their experimental evaluation, as well as the outcomes of these studies. Critical data analysis will contribute to greater methodological standardization and the consequent universalization of procedures to test NES activity.

## Laboratory methodological aspects

The possibility of applying NES directly to the infected wound represents a promising alternative that can solve problems such as the anxiety of patients and clinicians about using live larvae, especially due to the possibility of the larvae escaping from the wounds. In addition, manifestations of irritation, pain, itching, and hypersensitivity during treatment may be factors that hinder the direct use of larvae (26).

The evaluation of the antimicrobial activity of larval NES depends on the selection of optimal conditions for carrying out the tests, from obtaining the larvae and their extracts to the final stage of quantifying their potency. Although the *in vitro* antimicrobial activity of NES and/or its components has been well demonstrated, the literature has shown many contradictory results. This is due, for example, to assays with different degrees of sensitivity, variations in larval preparation, different conditions for obtaining and maintaining NES, use of total NES or its fractionated components, types and quantities of diluents used in the extraction, type of microorganism tested, and time of exposure to NES.

**Figure 2:**
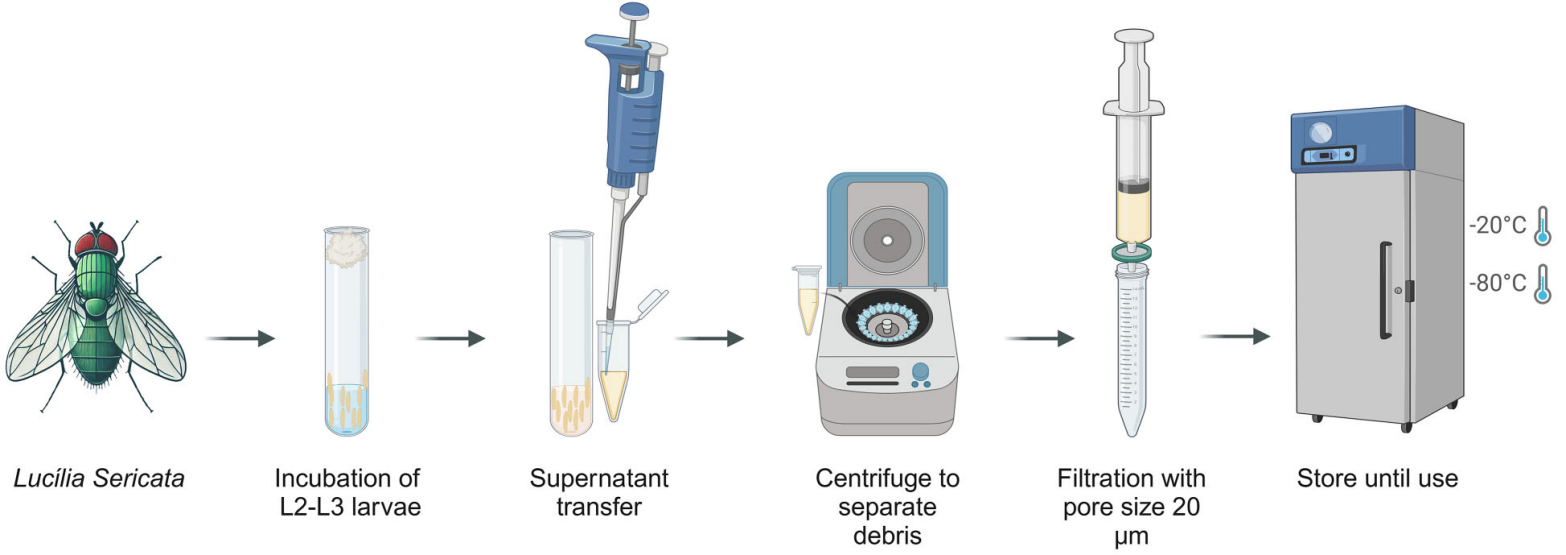
General scheme for collection, processing, and storage of native excretions and secretions (NES) from *Lucilia sericata.*

Therefore, it is urgent to standardize procedures so that the results are consistent and comparable. It is also important to define the spectrum of antimicrobial activity of NES. The following sections gather data on NES activity in relation to factors such as heating, pH, larval preparation procedures, NES extraction, and tests to evaluate the antimicrobial activity of NES. Figure 2 presents a general scheme for the collection of *Lucilia sericata* excretions and secretions. As incubation time and temperature may vary among different studies, the diagram illustrates only the main steps, from larval incubation to the storage of the collected material for future use.

### Antimicrobial and antibiofilm activity of heat-treated **NES**

Most reports show that heat treatment of NES does not result in loss of its antimicrobial activity. The thermostability of these larval excretions/secretions has been demonstrated at temperatures ranging from 100 to 121°C.

Simmons (27) developed the first studies to heat NES aiming at the sterilization of these excretions/secretions, since the larvae were obtained in a non-sterile way. Heat-treated NES (110°C for 20 min) from *Lucilia sericata* larvae maintained bactericidal activity against *Staphylococcus aureus*, *Streptococcus pyogenes*, *S. faecalis* (*Enterococcus faecalis*), *S. mitior*, *Clostridium welchii*, *Proteus vulgaris*, and *Eberthella typhi* (*Salmonella* Typhi). Likewise, Pavillard and Wright (28) observed that NES from *Phormia terraenovae* (*Protophormia terraenovae*) (Robineau-Desvoidy, 1830) larvae treated at 121°C for 30 min maintained bactericidal activity against *Streptococcus* group A, *pneumococci* type 1, *Streptococcus* group B, and *Streptococcus* viridans. About 40 years later, Thomas et al. (29) also reported that boiling NES from *Lucilia sericata* larvae did not alter its antimicrobial activity against *Streptococcus* groups A and B and *S. aureus*. Their results suggested that if an active agent was present, it was probably not an enzyme.

In agreement with the previous studies cited, Bexfield et al. (25) reported that the antibacterial activity of NES from *Lucilia sericata* against *S. aureus* was remarkably heat stable, with activity maintained at 100°C for 60 min. Interestingly, in the colony forming unit (CFU) assay, these authors also observed that incubating *S. aureus* with untreated NES in phosphate-buffered saline significantly increased the number of bacteria in just 30 min (123% compared to the control). In addition, in the same period, NES treated at 100°C for 8 min reduced the bacterial concentration to 23% of the control. The authors attributed this intriguing activity of heat-treated NES to the possible revocation of a specific inhibitor or the activation of the antibacterial factor itself in some way. On the other hand, an assay to evaluate the lytic activity of NES in the well-plate method showed cell lysis of the indicator microorganism (*Micrococcus luteus*) with potency comparable to that of 581 U/mg of lysozyme. This property was abolished by heating the NES for 5 min at 100°C. The authors concluded that the component(s) responsible for this lytic action would be proteases in the NES. Other thermostable components present in the secretions would be responsible for its antimicrobial activity. Given their thermal stability, these agents were considered non-proteinaceous and non-enzymatic factors lacking a complex structure.

Teh et al. (30) developed a new procedure to produce a whole-body extract of *L. cuprina* larvae via methanol extraction and showed that, after drying and reconstitution in water, the extract heated to 100°C/5 min or 121°C/20 min was shown to have potent antibacterial activity against a wide range of pathogenic Gram-positive and Gram-negative bacteria. In another line of investigation, Evans et al. (31) observed that NES from *L. sericata* larvae showed significant antifungal properties against different yeast species with a highly heat-stable component. Interestingly, the authors showed that this activity was retained at all investigated temperatures and increased significantly after treatment at 50°C for 15, 30, and 60 min. No difference was observed when heated to 100°C for 15 min. However, there was a significant increase in antifungal action after heating at 100°C for 60 min, indicating that the NES became more active after being treated at a higher temperature for a longer period. As mentioned above, it is important to consider that Bexfield et al. (25) also reported this feature for heat-treated NES against *S. aureus*.

In 2015, Ratcliffe et al. (32) reinforced the premise that the antibacterial components present in NES are thermostable. They preliminarily evaluated the larval excreta action of three calliphorid species against *Escherichia coli*. At least for *Chrysomya megacephala* (Fabricius, 1794), antimicrobial activity was maintained after heating for 1 h at 100°C. Similarly, according to El-Bassiony et al. (33), heating NES from *Sarcophaga argyrostoma* (Robineau-Desvoidy, 1830) in a water bath at 100°C for 5 min did not significantly interfere with its antimicrobial action against *E. coli* for a period of up to 18 h of incubation. Although incubation of heat-treated NES for 24 h led to a significant loss of potency against the microorganism, the same was observed for untreated NES.

Based on the studies reviewed above, there is strong evidence that antimicrobial components in NES remain active after warming. In opposition to these findings, Kerridge et al. (34) observed that boiling the larval extract of *L. sericata* resulted in a complete loss of antibacterial activity for methicillin-resistant *S. aureus* (MRSA). However, the authors pointed out the lower sensitivity of the agar diffusion assay as a possible explanation for this result when compared to the liquid culture assay used in previous studies.

In addition to studies showing the thermostability of NES activity, it is worth mentioning reports on the preservation of its antibacterial activity after freeze-thaw cycles (25,31-34), as well as resistance to proteases (25,32). These properties must be considered when discussing the need for methodological standardization for studies or applications of NES.

In another approach, some studies have evaluated whether the antibiofilm activity of heated NES remains the same as that of normal NES. Biofilms are complex multicellular communities of microorganisms that proliferate surrounded by a polymeric matrix, providing protection against antimicrobial drugs and the host's immune defense (35). The treatment (2-h boiling) of the larvae NES of *L. sericata* abolished its antibiofilm activity against *S. aureus*, both for biofilm formation and the established biofilm. However, for *P. aeruginosa*, heat treatment did not affect NES antibiofilm activity, either regarding biofilm formation or mature biofilm, suggesting an action in which different molecules would be responsible for the observed effects (36) or differences in the chemical composition of the respective biofilms. In other words, heat-sensitive proteins or peptides present in NES could provide antibiofilm activity for one microorganism but be inactive for others. Purifying these compounds would be a way to better understand the mechanisms involved in the modulatory effects of NES in biofilms.

According to Harris et al. (37), larval NES (not-heat treated) of *L. sericata* significantly inhibited nascent biofilm formation and disrupted preformed biofilms produced by two strains of *S. epidermidis*. The percentages of reduction in biofilm formation for the respective strains were 71.0 and 93.5% compared to control. Meanwhile, the disaggregation values for preformed biofilms were 79.8% and 94.2%. When testing NES treated at 100°C for periods of 5 to 60 min, an antibiofilm effect was also observed, but with less pronounced results compared to the unheated NES, on nascent as well as mature biofilms. For example, NES boiled for 5 min lost 31 and 9% of the inhibitory activity on nascent biofilms and 31 and 56% of the ability to disaggregate mature biofilms compared to untreated NES. Longer NES heating times resulted, with some fluctuations, in decreased inhibitory activity of biofilm formation for the strains, but without being completely abolished even after heating for 60 min. However, regarding mature biofilms, disaggregation was abolished by boiling NES for 10 min. The authors suggested enzymatic components in NES to be responsible for antibiofilm activity.

Bohova et al. (38) also obtained varied results when comparing the activity of normal NES and heat-treated NES (100°C/10 min) of *L. sericata* on biofilm formation and mature biofilm of three wound isolates (*S. aureus*, *Enterobacter cloacae*, and *P. mirabilis*). While NES significantly inhibited biofilm formation in *S. aureus* and *E. cloacae* and stimulated biofilm formation in *P. mirabilis*, heat-treated NES did not affect biofilm formation in *E. cloacae* and *P. mirabilis*. However, it reduced biofilm formation by 51% in *S. aureus*. Regarding mature biofilm, unlike NES, heat-treated NES did not promote the disaggregation of *E. cloacae* and *S. aureus*. Only for *E. cloacae* did NES show significant lethal activity on cells within the biofilm. Nevertheless, for heat-treated NES, this activity was lost. Tests performed with protease (proteinase K) showed that the antibiofilm activity for *E. cloacae* and *S. aureus* was comparable to that observed for NES. Furthermore, NES (but not heat-treated NES) showed proteolytic activity in tests with milk agar plates. Therefore, these data indicate that the antibiofilm activity of NES varied according to the composition of the biofilm produced by the microorganisms, and this activity would be more associated with the presence of proteases. These enzymes probably have a high molecular weight and complex structure, being degraded when NES is heated. Although not discussed by the authors, the significant inhibition of heat-treated NES for biofilm formation in *S. aureus* may suggest the presence of some thermostable component that does not act on the mature biofilm. General data on the antibiofilm effect of larval excretions/secretions are reviewed in Morris et al. (39).

### pH-related NES effects

Investigations that mention the pH of NES have not established a direct correlation of this physicochemical variable with antimicrobial activity. In a study by Baer in 1931 on the treatment of chronic osteomyelitis, republished in 2011 (4), the author observed that the pH of wound fluids became alkaline on the second or third day after starting maggot therapy. He believed that this change would contribute to reduced bacterial growth. This alkalinization would result from the secretion of ammonia by larvae and its derivatives, which would inhibit bacterial growth and act on wound healing (40-42). Subsequently, Chambers et al. (43) showed that alkaline pH is necessary for the activity of proteolytic enzymes (serine proteases and metalloproteinase) present in NES of *Lucilia sericata*. Moreover, it is important for degrading fibrin, fibronectin, laminin, and other extracellular matrix components in wounds. These enzymes could influence wound healing events when larvae are introduced into necrotic and infected wounds, with a chymotrypsin-like activity involved in remodeling extracellular matrix components. This study found that *L. sericata* larvae increased the pH of distilled water from 6.0-6.5 to >7.5 *in vitro*. However, it did not investigate the antimicrobial activity of NES.

Thomas et al. (29) published one of the first studies on the antimicrobial activity of NES that mentions the pH factor more specifically. After verifying that NES produced by *Lucilia sericata* larvae had a pH of 8.0-8.5, these data were used to adjust the pH to different values in liquid cultures of Gram-positive and Gram-negative bacteria without adding NES. Cultures were monitored for 5 h through absorbance readings. The authors preliminarily concluded that the pH value alone was not responsible for the inhibitory effects of NES in the control cultures tested.

More extensive data on the influence of pH on the antimicrobial activity of NES were presented by Bexfield et al. (25). When evaluating a large number of NES samples (n=91) from sterile *L. sericata* larvae, they found that the pH ranged from 7.6-9.0, the majority being 8.2-8.3. Then, NES samples with pH≥8.0 were adjusted to pH values 6.0 and 7.0 and assayed to *S. aureus* using the turbidimetric assay. However, these preparations retained significant antibacterial activity at pH 6.0 and 7.0, thus suggesting that this activity did not depend on alkaline conditions. Although the authors did not analyze this, it is possible to speculate that, in addition to the substance(s) with antimicrobial activity present in NES, a substance with alkaline pH could be secreted together so that pH neutralization did not affect antimicrobial activity.

Similarly, Cazander et al. (20) used the turbidimetric test to assess the antimicrobial activity of NES obtained from sterile *L. sericata* larvae on Gram-positive and Gram-negative bacteria. The NES obtained had a pH of 8.0. However, this investigation did not allow comparisons of the pH variable with other studies since, contrary to expectations, there was no evidence supporting the existence of bactericidal or bacteriostatic activity of larval secretion in the controlled *in vitro* experiments that were used. The absence of the antibacterial effect was also observed in tests with live maggots, and the authors hypothesized that, *in vivo*, there could be an indirect antibacterial mechanism in which the immune system is involved. Negative results for the association of pH with antimicrobial activity for NES from larvae of different insects were also reported by Barnes et al. (44). This study tested NES identified as alkaline (*L. sericata* [pH: 8.67-8.82] and *Calliphora vicina* (Robineau-Desvoidy, 1830) [pH: 8.53-8.68]) and acidic (*Dermestes maculatus* (DeGeer, 1774) [pH: 6.00-6.14] and *Tenebrio molitor* (Linnaeus, 1758) [pH: 5.14-5.28]) against *S. aureus*, *Bacillus cereus*, *E. coli*, *P. aeruginosa*, and *P. mirabilis* using the viable count assay.

Based on the above studies, we concluded that the pH of NES produced by larvae of fly species such as *L. sericata* and *C. vicina* are alkaline, ranging from 7.6 to 9.0. As pointed out by Messer and McClellan (40), this may be due to the presence of substances such as ammonia and its derivatives. However, the evidence does not make it possible to establish a direct relationship between the alkaline pH of NES and its antimicrobial activity.

### Larvae preparation, NES extraction, and standardization procedures

As shown in Table 1 (20,21,24,31,36,45,46) and Table 2 (32-34,38,44,47-56), there are variations in larval stage used, type of extractor used to obtain NES, number of larvae, extraction conditions, and treatment of the extract obtained. This lack of standardization makes comparisons between studies difficult. In order to contribute to standardization, we suggest a set of procedures in Table 3 (25,29,32,34,36,47,57,58) for larval selection, extraction, treatment, and evaluation of the antimicrobial activity of NES. The methodological data presented in this table are based on more efficient results reported in the literature.

**Table 1:** Native excretions and secretions (NES) extraction conditions using sterile commercial larvae for maggot therapy.

Reference	Larval stage	Extractor type	Quantity of larvae/extractor volume	Extraction conditions	Extract treatment
(20,21)	1st and 3rd	Saline solution (0.9%)	L1: 400 larvae/100 µL L3: 200 larvae/100 µL	1 h at 35°C in the dark	Adjusted to 675 µg/mL and serially diluted in PBS
(24)	1st and 3rd	Milli-Q UW	1 g/200 µL	1 h at 30°C in the dark	CF
(31)	1st, 2nd, and 3rd	Milli-Q UW	1 g/200 µL	1 h at 30°C	CF
(36,45)	2nd and 3rd	Milli-Q UW	50 larvae/200 µL	1 h at room temperature in the dark	CF
(46)	1st and 2nd	Milli-Q UW	L1: 300 larvae/200 µL L2: 400 larvae/200 µL	1 h at 30°C in the dark	CF

Milli-Q UW: Milli-Q^®^ ultrapure water; CF: centrifugation; PBS: phosphate-buffered saline. Larval stages 1st, 2nd, 3rd: egg, larva, pupa.

**Table 2:** Native excretions and secretions (NES) extraction conditions using own-production larvae.

Reference	Larval		Extractor type	Quantity of larvae/extractor volume	Extraction conditions	Extract treatment
	Stage	State				
(32)	Final stage	Prior fasting of 18-24 h	Milli-Q UW	1 g/100 µL	1 h at 37°C	CF+SF
(33)	3rd	Decontamination with formaldehyde and alcohol	PBS 7.2	200 larvae/2 mL	1 h at 27°Cin the dark	CF+SF
(34)	2nd and 3rd	Decontamination with 70% ethanol	ddH_2_O	100 larvae/10 mL	Overnightat 25°C	CF+SF+LP
(38)	3rd	Not sterile and sterile	Milli-Q UW	50 larvae/200 µL	1 h at 4°C	CF+LP
(44)	3rd	Not sterile	diH_2_O	1 to 4 g/1 mL	1 h at 30°C	CF+SF
(47)	NC	Not sterile	dH_2_O	150-200 g/50 mL	30 min	CF+SF+LP
(48)	NC	NC	Milli-Q UW	10 g/4 mL	Overnightat 25°C	CF+SF+LP
(49)	3rd	Decontamination with formaldehyde and hypochlorite	PBS	200 larvae/2 mL	1 h at 25°C	CF+SF
(50)	3rd	Decontamination with formaldehyde and hypochlorite	SS	≅1500 larvae/6 mL	1 h at 37°Cin the dark	CF+SF
(51)	2nd and 3rd	Obtained from sterile eggs	dH_2_O	50 larvae/1 mL	Rinsingfor 4×	SF
(52)	72 h feeding	Decontamination with alcohol	dH_2_O	25 larvae/800 µL	1 h at 37°Cin the dark	AT (121°C/20 min)
(53)	2nd and 3rd	Sterile	SS	NC	1 h at 37°Cin the dark	CF+SF+LP
(54)	3rd	Decontamination with alcohol	diH_2_O	100 larvae/200 µL	1 h at 30°C	SF
(55)	Before pupal stage	Sterile	dH_2_O	160 larvae/50 mL	NC	SF
(56)	3rd	Decontamination with alcohol	PBS	100 larvae/200 µL	6 h at 25°Cin the dark	CF+SF

Milli-Q UW: Milli-Q^®^ ultrapure water; ddH_2_O: double-distilled water; dH_2_O: distilled water; diH_2_O: deionized water; SS: saline solution; CF: centrifugation; SF: sterilizing filtration; LP: lyophilization; AT: autoclaving; NC: unspecified. Larval stages 1st, 2nd, 3rd: egg, larva, pupa.

**Table 3:** Suggested protocols for obtaining native excretions and secretions (NES)/evaluation of antimicrobial activity.

Step	Procedures	Justification (reference)
1. Selection of larvae	Species: *Lucilia sericata* (L3)	L3 larvae produce a greater volume of NES with greater antimicrobial activity (25)
	Decontamination: 70% ethanol (2 min) + 1% hypochlorite (5 min) + Milli-Q water	Eliminates external microbiota without impairing NES activity (34)
	Fasting: 18-24 h before extraction	Reduces intestinal waste, resulting in clearer and more concentrated NES (32)
2. NES extraction	Ratio: 1 g of larvae to 200 µL of Milli-Q water	Maximizes the concentration of active components (25)
	Incubation: 30°C for 1 h in the dark, with gentle shaking (50 rpm)	Preserves heat-sensitive compounds such as proteases (29)
	Post-processing: centrifugation (10,000 *g*, 5 min) + filtration (0.22 µm)	Removes debris and ensures sterility (25)
3. Treatment of NES	Fractionation: ultrafiltration filters (10 kDa → 500 Da)	It isolates the fraction <500 Da, which contains thermostable dipeptides with high activity (57)
	Lyophilization: freeze-dry and reconstitute in Milli-Q water (10×)	Preserves antimicrobial activity and allows storage (47)
	Heating: treat at 100°C for 60 min	Inactivates proteases and concentrates thermostable compounds (25)
4. NES evaluation	Turbidimetric method: inoculate 1×10^6^ CFU/mL in TSB + NES (25, 50, 75%)	Evaluates the kinetics of microbial growth inhibition (58)
	Flow cytometry: stain with Syto-9 (viable) and propidium iodide (dead)	Detects cells in VBNC state (57)
	Biofilm: treat preformed biofilms with NES for 24 h	Assesses the dissolution of biofilms (36)
5. Storage	Fractionated and lyophilized NES: store at -80°C, in aliquots	Maintains antimicrobial activity for months (57)

TSB: tryptic soy broth; VBNC: viable but non-culturable; CFU: colony forming units.

In the initial studies, the procedures for extracting (NES comprised the following steps: applying large amounts of eggs to raw or decomposing meat; feeding the larvae for three days; transferring the larvae to a sieve placed over a funnel attached to the neck of a flask; and spraying with water for 10 min to 4 h. Then, the drained liquid was collected and heat-sterilized for use in antimicrobial activity tests (27,28).

Subsequently, Thomas et al. (29) employed centrifugation of the washing liquid to remove particulate material, which was frozen until testing. A few years later, in a variation of this method, Bexfield et al. (25) introduced new and important modifications for the extraction of NES, which remain the basis of the methodologies used in most studies. These authors used commercially available sterile larvae for maggot therapy; the larvae were placed in a container with sterile ultrapure water at a rate of 200 µL per gram of larvae. After incubation for 1 h at 30°C in the dark, the sterile liquid was siphoned from the containers and centrifuged at 10,000 *g* for 5 min to remove particulate material. Then, the supernatant was retained for antimicrobial activity tests and characterization of NES components. Tables 1 and 2 summarize the larval preparation/NES extraction conditions used by Bexfield et al. (25) and the variations of this technique that were later introduced by other authors, relating to the larval instar used, the procedures for obtaining sterility of the larvae, the proportion/quantity of larvae and extracting liquid, and the treatment used in the newly obtained NES.

**Figure 3:**
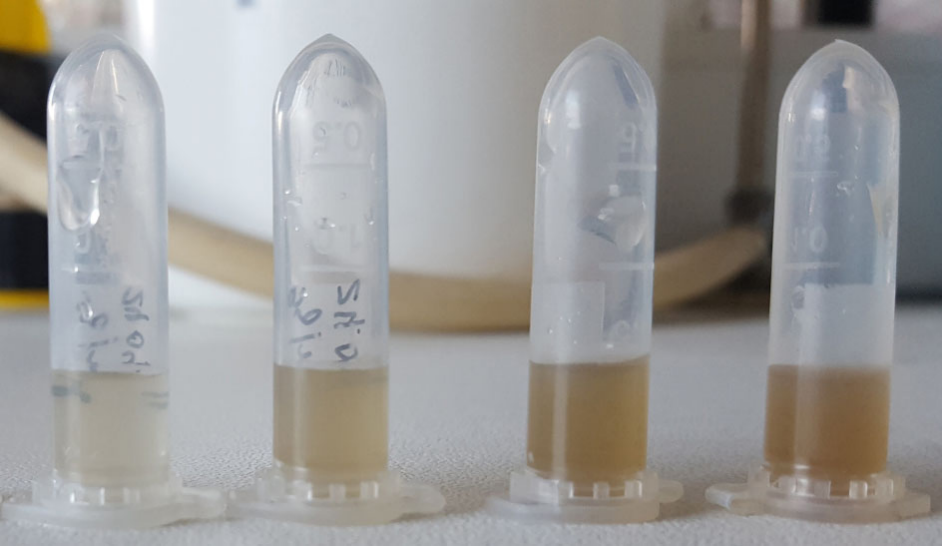
Native excretions and secretions (NES) with progressively dark brown color resulting from the larvae droppings.

Among the studies cited in Table 2, we highlight the modification proposed by Ratcliffe et al. (32), where final-stage larvae were collected and starved for 18-24 h before washing with sterile Milli-Q. For NES extraction, 1 g of larvae was weighed and mixed with 100 µL Milli-Q UW in 200 mL polystyrene flasks and incubated for 1 h at 37°C. According to Kruglikova and Chernysh (47), there are many impurities, including pigments, in larval secretions. Therefore, even at a high NES concentration (i.e., using a minimum volume of the extractor for a given weight/number of larvae), the exosecretion contains a small number of antimicrobial components. Thus, we consider that larval fasting significantly reduces the presence of intestinal contents of larvae in the NES, reducing the presence of these residues when extracting the NES. Therefore, this procedure makes it possible to obtain a clear supernatant after centrifugation without the dark brown color resulting from the larvae droppings, as shown in the images in Figure 3 generated in our laboratory. Obtaining clear and colorless NES is important when it is submitted to spectrophotometric methods or protein dosage.

In order to improve NES performance in antimicrobial activity tests, Kruglikova and Chernysh (47) extracted the hydrophobic fraction of larval excretion/secretion using Waters Sep-Pak Vac 20cc cartridges with C18 sorbent, followed by lyophilization. Lyophilization, either of fractions or of total NES, was used in different studies. It provides an important means of concentrating the antimicrobial products present in NES. In addition, it allows the standardization of secretions between naturally variable batches of larvae. The larval extract withstands lyophilization well, and the reconstituted secretions can withstand several freeze-thaw cycles. Furthermore, the lyophilized product can be stored for a long time, resulting in no detectable loss of subsequent antibacterial activity (34).

Another factor to be considered in the standardization of NES for assays of antimicrobial activity is the presence of proteases, whose activity is provided by the alkaline pH of the NES due to the presence of ammonia and its derivatives (40). Pickles and Pritchard (59) proposed the previous evaluation of the proteolytic activity of NES on gelatin agar by a “radial diffusion enzymatic assay” as quality control for NES of commercially available sterile larvae for maggot therapy. The proteolytic action of NES was also evaluated using skim milk agar plates and proteinase K as a positive control (38), with loss of NES proteolytic activity occurring after heat treatment. Bexfield et al. (25) reported a similar result showing the lytic action of NES on *Micrococcus lysodeikticus* (*luteus*) lyophilized in the agar-well diffusion method.

For investigators who use unfractionated or non-lyophilized NES in antimicrobial activity tests, a useful standardization alternative is to measure the amount of protein present in the larval extract. Among the available techniques, the Bradford assay is simple and highly sensitive. It is used extensively to determine the concentration of proteins in a wide variety of samples. The assay is based on the binding of the Coomassie blue dye to the protein(s), resulting in a dye-protein complex with higher molar absorbance. The reading in this assay is done at a wavelength of approximately 595 nm (60). Peptides with a molecular weight lower than 3000 Da do not form a complex with the dye, making their reading and consequently their quantification by this method unfeasible (61). Bexfield et al. (25) performed the molecular fractionation of NES and observed greater activity in the <500 Da fraction.

However, despite reports of low molecular weight dipeptides in NES, most of the peptides identified have a size greater than 3000 Da. Furthermore, the NES constitutive proteases mentioned above are high molecular weight proteins, and they are also detected in this assay.

### NES molecular fractionation

An important factor when discussing the optimization of maggot therapy is the identification of the components of NES that are responsible for its antimicrobial activity. An obvious way to answer this question is to use fractionation techniques to isolate such molecules to allow their chemical characterization and a better understanding of their properties.

Bexfield et al. (25) presented one of the first reports on molecular mass fractionation of NES using an Amicon stirred ultrafiltration cell pressurized with N_2_ gas. Based on a preliminary investigation that indicated the presence of an antibacterial factor with a molecular weight <3 kDa, the authors sequentially used Amicon filters with a molecular weight cut-off (MWCO) of 10 kDa and 500 Da to generate fractions above and below the cut-off. Thus, the fractionation of *L. sericata* NES yielded three molecular mass fractions (>10 kDa, 500 Da-10 kDa, and <500 Da). The <500 Da filtrate, including MRSA, demonstrated significant antibacterial activity against *S. aureus* in the TB assay. Meanwhile, the 500 Da-10 kDa fraction showed significant antibacterial activity against *S. aureus* but not against the MRSA strain. The retentate >10 kDa showed no activity against *S. aureus*. However, it had a lytic action on *M. lysodeikticus* cells in the zone of clearance assay originally used to determine lysozyme activity. The impairment of lytic activity with the heat treatment and the lack of lytic activity in the lysozyme assay were attributed to the probable presence of abundant proteases in the larval NES. The antibacterial activity of NES was not affected by heating, trypsin, and pronase E. Furthermore, the authors concluded that the activity observed in the <500 Da fraction was not due to antimicrobial peptides from known insects (magainins, apidaecins or defensins), as their molecular mass would be 2 to 4 kDa. Possibly, this action would be related to the presence of antimicrobial dipeptides such as those described in larvae of the dipterans *Neobellieria bullata* (Parker, 1916) and *Sarcophaga peregrine* (Robineau-Desvoidy, 1830), which are also resistant to heat treatment and to the action of proteases.

Four years later, following the same methodology described in the 2004 study, Bexfield et al. (57) used sequential molecular fractionation of the NES from *L. sericata* to characterize the antibacterial effects of the <500 Da fraction (ES_<500_) on a variety of bacteria, including 12 strains of MRSA. The ES_<500_ induced distinct morphological changes in *B. cereus* and *Escherichia coli*, bacteriostatic activity for *S. aureus*, and bactericidal activity for *E. coli*. In addition, it is a feasible but non-culturable state in this Enterobacteriaceae. *Bacillus subtilis* and *Klebsiella pneumoniae* were the most sensitive to the antibacterial effects of ES_<500_, while *P. mirabilis* and *S. epidermidis* were the most resistant. Furthermore, NES <500 Da significantly inhibited ten of the twelve MRSA strains tested, but unfractionated NES inhibited the two unaffected strains. Interestingly, however, storage of NES at -80°C instead of -20°C before ultrafiltration restored ES_<500_ activity against these strains.

Using sequential filtration on Amicon Centricons filters (MWCO 50, 30, 10, 5, and 3 kDa), Kerridge et al. (34) also studied the antibacterial activity and molecular size of compounds present in the NES of *L. sericata* larvae against an epidemic strain of MRSA. All filtrates obtained showed anti-MRSA activity, and the retentates of the 10 and 5 kDa MWCO filters indicated the presence of at least one additional larger antibacterial compound, distinct from the <3 kDa activity. Furthermore, the 3 kDa filtrate passed through a micropartition device fitted with a 1 kDa filter. It revealed the presence of anti-MRSA activity in the resulting filtrate, indicating the presence of at least one active compound <1 kDa. Sodium dodecyl sulfate electrophoresis using high-density gels designed for peptide separation revealed several low molecular weight bands as further evidence of low molecular weight antibacterial compounds in the crude larval extracts. When comparing their findings with those of other studies, the authors emphasized that antibacterial peptides smaller than 5 kDa belonging to the group of defensins, with activity mainly against Gram-positive bacteria, are not uncommon and were isolated from dipteran species such as *Sarcophaga peregrina* and *Phormia terraenovae*.

Compared to bacteria, the antifungal properties of larval NES are less well-known. Evans et al. (31) evaluated the effect of NES from *L. sericata* subjected to successive filtration with Amicon Ultra with molecular cut-offs of 10 kDa and 500 Da on *Candida albicans* SC5134, and other yeasts (*Saccharomyces cerevisiae*, *S. boulardii*, *C. krusei*, and *C. maltosa*). The colony forming unit (CFU) assay was used to detect the antifungal activity of NES and fractions on *C. albicans*. The absorbance assay was used for all species. The CFU assay demonstrated antifungal capabilities in NES, ES 10-0.5, and ES<0.5 kDa fractions, with the <0.5 kDa fraction showing the greatest activity level. Antifungal activity was not observed in retentate >10 kDa. On the contrary, there was an increase in CFU compared to the control. In the absorbance assay, incubation with ES fractions revealed a significant growth inhibition against all fungal species tested at the mid-log growth phase. The ES fraction <0.5 kDa also showed strong antifungal activity. The differences observed between the CFU and absorbance assays were attributed to the nature of the assays. While in the UFC assay, the fungal incubation in the presence of NES or its fractions is followed by seeding on the agar plate where these compounds are no longer present (by dilution), in the absorbance assay, the antifungal agents are present throughout the growth period. Thus, the absorbance assay seems to better demonstrate the kinetics of fungal growth.

Employing a more complex procedure for the fractionation of larval NES, Kruglikova and Chernysh (47) obtained hydrophobic compounds from the NES of *L. sericata*. After being extracted on Waters Sep-Pak Vac 20cc cartridges with C18 sorbent, NES was fractionated using high-pressure hydrophobic reverse-phase liquid chromatography (HPLC), chosen for its high efficiency in separating peptides. An Ascentis C18 column was used, and elution was carried out at the linear acetonitrile gradient. The chromatographic fractions obtained were vacuum-dried and dissolved in distilled water, and their antibacterial activity was tested by the agar well diffusion method. The fraction corresponding to 30 and 31% of acetonitrile showed inhibitory action on *M. luteus*, while the fraction produced at 32-34% inhibited *E. coli*, indicating that the components responsible for the activity against Gram-positive and Gram-negative bacteria can be completely separated by the HPLC method. Mass-spectrometry with electrospray ionization (ESI) and matrix-assisted laser desorption ionization time-of-flight (MALDI-TOF) were used to qualitatively analyze the compounds. The active compounds against *M. luteus* have low molecular weight compounds with masses from 129 to 700 Da and two peptides with masses of 6466 and 6633 Da. The low molecular weight compounds with activity against *E. coli* presented masses of 174 to 904 Da, while the peptides presented molecular masses of 1014, 1128, 1269, 5772, 8631, 8882, and 9025 Da.

A previous study by Chernysh et al. (62) identified defensins against Gram-positive bacteria in chromatograms of *Calliphora vicina* hemolymph, and classes of diptericins and cercopins against Gram-negative bacteria, which were eluted at 30-35% acetonitrile. Interestingly, Kruglikova and Chernysh (47) showed that mass spectrometry revealed polypeptides with molecular masses (8882 and 9025 Da) in the hemolymph of *L. sericata* almost identical (8886.2 and 9029.1 Da) to those described by Chernysh et al. (62). The nature of the low molecular weight components has not been identified. Kruglikova and Chernysh (47) suggested that these compounds may be secondary metabolites functionally similar to antibiotics of microbial origin.

### Tests to assess the antimicrobial activity of NES

Although the methods currently used to determine the antimicrobial activity of NES are very diverse, they are more or less related to the conventional tests used to determine bacterial susceptibility to antimicrobials. However, unlike the latter tests, which follow strict recommendations from standards developed by international committees, such as the CLSI (Clinical and Laboratory Standards Institute), there is still no standardized universal methodology for detection, quantification, and proof of the antimicrobial efficacy of compounds present in larval secretions/excretions. Variations in experimental procedures are probably the cause of the discrepancies in the results reported by different authors, making comparisons difficult.

In addition to the disparities in the procedures used to obtain and maintain the NES, factors that can influence the sensitivity and reproducibility of the methods include the type and nutritional richness of the culture medium used, the concentration of the active antimicrobial component present in the NES, the size of the microbial inoculum, the non-availability of reference microorganisms with well-characterized susceptibility or resistance to NES for the quality control of the tests, the growth stage of the inoculated microorganism, and the incubation time stipulated for the tests. In addition, there are factors such as the type of extractor of the NES used, whether lyophilized extract was used or not, and whether crude or purified NES extract was used after molecular fractionation. Moreover, the larval stage must also be considered to obtain the NES. For example, using the TB assay, Bexfield et al. (25) reported that first-stage larval NES showed significant antibacterial activity against *S. aureus* and *E. coli*. Meanwhile, third-stage larval NES showed higher yields and significant antibacterial activity against many potential Gram-positive and Gram-negative pathogens. On the other hand, Barnes et al. (44) emphasized that the number of larvae used must be sufficient to produce significant antibacterial activity, since some components present in NES are also used for bacterial growth.

The most commonly used techniques to evaluate the antimicrobial activity of NES include: i) turbidimetric assay; ii) agar-well diffusion method; and iii) determination of the number of viable bacteria. These techniques have been used alone or in combination according to the study's objectives. A brief description of the fundamentals of these techniques is given below.

#### Turbidimetric assay

This method consists of adding different concentrations of NES in a liquid culture medium and inoculating a standardized number of the test microorganism. After the established incubation period, the reading is performed based on the reduction of turbidity compared to the control medium without NES inoculated with the test microorganism. Although this procedure does not allow numerical differentiation between viable and non-viable microbial cells, the effect of NES on the target microorganisms can be monitored kinetically during the test period (29). Furthermore, turbidimetric assays can be complemented by counting viable bacteria on solid media.

According to Rippere (58), the turbidimetric tests present greater accuracy and precision than those performed on agar. However, it is important to consider appropriate nutritional conditions in liquid culture assays. Barnes et al. (44) indicated that the addition of 10% tryptone soy broth (TSB) to NES, whilst prolonging the time required to achieve a reduction in bacterial cell viability, demonstrates the actual antibacterial potency of NES over a 24-h period. An incubation period of 24 h appears most appropriate as it allows a lag phase or inhibition period to become apparent in turbidimetric studies. On the other hand, according to the authors, a very nutrient-rich medium can negatively affect the antimicrobial activity of NES. Furthermore, the antibacterial activity in NES is continuously produced and might therefore be considered more effective *in vivo* than when used alone *in vitro*.

#### Agar-well diffusion method (sometimes called zone of inhibition assay)

This method is based on mixing an aliquot of the test microorganism in a solid culture medium previously melted and cooled to 50°C, homogenization, and plate distribution. Then, perforations are made in the center to obtain holes of standardized diameter. The holes are loaded with standardized volumes of NES dilutions, and after incubation, the reading is performed by measuring the diameters of the inhibition halos formed around the holes (25,34,47). In a variation of this method, sterile filter paper discs are impregnated with NES solutions containing estimated concentrations of proteins, dried, and added to the surface of the medium previously seeded with the test microorganism. In this method, the reading is done by measuring the diameter of the inhibition halo around the disc, similar to the disc-diffusion test traditionally used to determine susceptibility to antimicrobials (33,48). Another variation of the technique is to add a nutrient-poor culture medium containing 1% agarose and the standardized inoculum of the bacterial culture to the plate. After solidification, aliquots of the different concentrations of NES are placed in the wells, and the plates are incubated for a short period for diffusion of the NES. Then, the plate medium is covered with a layer of the liquid culture medium containing agarose. After incubation, the inhibition halos' diameters are measured (49).

#### Colony-forming unit (CFU) assay and other methods

The CFU count method is classically used for several purposes, such as to evaluate substances with antimicrobial action or to quantify microorganisms in products like water, food, cosmetics, or even in clinical specimens such as urine. The investigation of the antimicrobial activity of NES by this method comprises the exposure of the larval extract to a standardized value of the microorganism for a certain time, followed by plating in the culture medium using the spread plate or pour plate technique. After incubation of the plates, the reading is done by counting the colonies and then comparing the final result with the number of microorganisms in the original inoculum.

## Traditional and other methods for assessing NES activity

The first laboratory procedures that made it possible to obtain evidence on the antimicrobial action of NES were described in the 1930s and 1950s, which we briefly describe for reasons of historical importance. Simmons (27) inoculated suspensions of *S. aureus* in aqueous solutions containing autoclaved excretions of *L. sericata* larvae. Then, aliquots of these suspensions and their dilutions were plated after varying exposure times. In parallel, aliquots of 5×10^6^ cells of *S. aureus* and six other bacterial species (24 h cultures in liquid medium) were added to solutions containing larval excreta, followed by inoculation in broth and plating at specific time intervals. The experiments were rated as absence of bacterial growth, light growth, or heavy growth. The author observed that, in general, short exposures to larval excreta promoted a lethal action on bacterial isolates.

Later, Pavillard and Wright (28) used the bacterial overlay technique to show that secretions of *Phormia terraenovae* larvae had a bactericidal effect against *Streptococcus pyogenes* and *Streptococcus pneumoniae*. On the other hand, *S. aureus* and *Clostridium welchii* were much less sensitive, while *E. coli*, *P. vulgaris*, *S.* Typhimurium, *B. subtilis*, and *C. albicans* were all highly resistant. In this study, solutions obtained by washing the larvae with distilled water were sterilized by heating and diluted in a fluid culture medium. In the next step, the authors isolated the antibiotic fraction in the wash with a chromatogram, using strips of Whatman No. 1 chromatography paper containing a solution of p-toluene sulphonic acid in n-butanol previously saturated with 2 percent saline. After drying and sterilizing by ultraviolet light, the chromatogram was embedded in agar, and *S. aureus* was streak-seeded in the longitudinal direction of the strips. After incubation, a marked zone of inhibition was noted at the upper end of the chromatogram. Relatively pure samples of the antibiotic fraction were obtained by washing the larvae using a cellulose column and modification of the chromatogram technique. However, the authors did not chemically identify this antimicrobial agent.

Since then, several studies have been conducted demonstrating the antimicrobial action of NES. However, many contrasting results have also been reported. Some results are even apparently paradoxical, such as the activity of NES promoting greater bacterial growth.

Bexfield et al. (57) combined turbidimetric assays and CFU counting with flow cytometry analyses to investigate the antibacterial activity of NES and a fraction <500 Da (ES<500 Da) of *L. sericata* larvae excretions/secretions against *S. aureus* and *E. coli*. The authors started from the premise that the traditionally used technique of determining bacterial viability through the formation of colonies on agar plates is very simplistic and does not consider that there are situations in which viable but non-culturable bacteria may be present. The preparations were treated with Syto-9 and propidium iodide (PI) for flow cytometry analysis. Syto-9 is a green fluorescent dye that labels nucleic acids regardless of whether the bacterial cell is alive or dead. PI is a red fluorescent nucleic acid dye that can only penetrate bacteria with compromised membranes, selectively labeling non-viable cells. Appropriately sized cells were then analyzed for fluorescence to determine bacterial feasibility after treating both strains with NES and ES<500 Da. For *S. aureus*, TB assay and CFU results showed significant growth inhibition after treatment with NES and ES<500 Da. In addition, flow cytometry revealed that most treated cells were still viable, suggesting a bacteriostatic effect detected by all three methods. For E. *coli*, the three methods also indicated a bacteriostatic effect of NES, but a bactericidal effect for ES<500 Da. This extract nearly abolished colony formation in the CFU test, reducing the initial bacterial inoculum by 100-fold, and flow cytometry revealed only 54% viability (compared to 85% viability for strain incubated with NES). Furthermore, interestingly, for *E. coli*, the phenomenon of induction by ES<500 Da of the viable but non-culturable state was observed. Based on the methodological aspects described in this study by Bexfield et al. (57), adding a vital dye to the TB assay to assess bacterial viability may be a less expensive alternative to flow cytometry, as performed by Fonseca-Muãoz et al. (50), who used triphenyltetrazolium chloride (TTC). The *in vitro* antimicrobial action of NES was complemented in some studies by an *in vivo* test. For example, after observing a bactericidal effect of secretions of larvae of *Protophormia terraenovae* on *Streptococcus* spp. in a fluid medium, Pavillard and Wright (28) isolated and partially purified the antimicrobial fraction, which was used for intraperitoneal inoculation in mice. This fraction was shown to protect these animals from the lethal effects of pneumococcal infection.

On the other hand, after *in vitro* evaluation (CFU count) of the activity of NES from *L. sericata* larvae against bacterial strains that frequently occur in chronically infected wounds, Jaklič et al. (51) evaluated the effect of applying sterile larvae on chronic wounds of thirty patients. Furthermore, bacteria isolated directly from non-sterile larvae were identified, typed, and tested for *in vitro* resistance to NES from these larvae. The 16S rRNA sequence similarities between the larval isolates showed that most of the resistant isolates were closely related to representatives of the genus *Providencia* and its relatives, while the remaining belonged to the genera *Proteus* (*P. vulgaris* and its relatives) and *Pseudomonas* (*P. fluorescens* and its relatives). Surprisingly, direct incubation of the maggot excretions/secretions resulted in isolating one Gram-positive strain, *Vagococcus lutrae*. Sterile larvae were administered to wounds inside a cage dressing (24 to 60 h), according to the procedure described by Sherman (63). Smears from the wounds and larvae were taken separately before and after every maggot application and placed in aerobic and anaerobic transportation media. The microorganisms were identified, and the antimicrobial activities were investigated by comparing the bacterial diversity in wounds before and after the application of *L. sericata* larvae. The application of larvae was considered efficient in 83.2% of cases, with 6.6% of the treated wounds completely healed and 76.6% of the wounds free of infection by pathogenic bacteria. After applying the larvae, the total number of species decreased in 63.6% of the analyzed wounds. Nine bacterial types originally present in the wounds were completely removed after application of sterile maggots: group *G streptococci*, group *C streptococci*, *B. fragilis*, *C. freundii*, *Klebsiella* spp., *Peptococcus* sp., *Prevotella bivia*, *Serratia marcescens*, and *S. agalactiae*. Considerable reduction was also observed in the incidence of 5 species: coagulase-negative staphylococci, *C. koseri*, *K. oxytoca*, *P. aeruginosa*, and *S. aureus.* Significant changes were not found *for K. pneumoniae*, *P. mirabilis*, *P. vulgaris*, *Stenotrophomonas maltophilia*, α-haemeolytic *Streptococcus*, and *S. pyogenes*, while surprisingly, six species increased in occurrence compared to the pre-treatment situation: diphtheroids, *Enterococcus faecalis*, *Morganella* sp., *Peptostreptococcus asaccharolyticus*, *Porphyromonas* sp., and *Providencia rettgeri*. The authors concluded that maggot therapy is highly recommended, but bacterial diversity in infected wounds is one of the factors affecting the success of this therapy. This type of therapy is most appropriate in infections caused by anaerobic and Gram-positive bacteria, and special precautions are needed when wounds are infected with certain Gram-negative bacterial species.

## Conclusions

Success in determining the *in vitro* antimicrobial activity of larval excretions/secretions depends on many factors, starting with larval preparation and the NES extraction technique. Despite certain variations in the procedures performed in different studies, the standardizations proposed in 2004 by Bexfield et al. (25) for NES extraction still remain a methodological basis in most investigations. One challenge is to obtain the material without the dark brown color that the extract naturally has, with many impurities, including pigments, which can influence the concentration and activity of the antimicrobial substances present. Reduction of impurities in the extract can be achieved by fasting the larvae for 18-24 h before washing, as proposed by Ratcliffe et al. (32), making it possible to obtain a clear NES. This procedure is also important in protein dosage studies to perform antimicrobial activity tests.

Although there is no evidence to establish a direct relationship between the alkaline pH of NES and its antimicrobial activity, it is also important to standardize this factor since the alkaline pH of NES provides the activity of the proteases present due to the presence of ammonia and its derivatives. Ideally, the pH should be close to 8.0 and it is important to avoid variations that are observed in the results in different studies, for example, due to the use of buffered extractants. Furthermore, the high pH of the NES could be used as an indirect indicator of the greater presence of compounds with antimicrobial activity since these compounds would be secreted together with alkaline substances. The analysis of the proteolytic activity could also be used as a parameter to evaluate NES since the proteolytic enzymes serine proteases and metalloproteinases are important in the degradation of fibrin and components of the extracellular matrix present in wounds. However, it is necessary to consider that sterilizing NES by heating, despite preserving the activity of antimicrobial compounds, causes the inactivation of the enzymes present.

Although the effectiveness of the maggot therapy technique has already been approved by regulatory bodies such as the FDA, the use of NES or its antimicrobial components still requires further investigation and methodological standardization, as there are studies with contrasting results. Investigations into the chemical nature of antimicrobial substances that may be present in NES must be expanded and deepened, and factors such as fractionation and lyophilization of the fractions obtained need to be considered in standardization. Despite the difficulties in obtaining a large quantity of highly purified proteins from original organic sources, recombinant protein technology may be a way to solve this problem (64). It is also important that the techniques for evaluating the antimicrobial activity of NES and its components are better defined to achieve a quality similar to that which currently exists for antibiotic susceptibility techniques. Likewise, in order to better understand the effectiveness of NES as a therapeutic measure, tests with its direct application to lesions should be expanded, preceded by the characterization of the infecting microorganisms present and *in vitro* evaluation of their susceptibility to NES. The direct use of NES or its purified antimicrobial components instead of the application of larvae would represent a great advantage in the treatment of chronic wounds, as it would eliminate one of the main obstacles to the acceptance of maggot therapy by patients and even in the medical field, i.e., the need to apply fly larvae to a wound.
